# Virtual reality-based training for mental health staff: a novel approach to increase empathy, compassion, and subjective understanding of service user experience

**DOI:** 10.1186/s41077-022-00217-0

**Published:** 2022-07-19

**Authors:** Simon Riches, Hannah Iannelli, Lisa Reynolds, Helen L. Fisher, Sean Cross, Chris Attoe

**Affiliations:** 1 King’s College London, Department of Psychology, Institute of Psychiatry, Psychology & Neuroscience, SE5 8AF London, UK; 2 King’s College London, Social, Genetic & Developmental Psychiatry Centre, Institute of Psychiatry, Psychology & Neuroscience, London, SE5 8AF UK; 3 South London and Maudsley NHS Foundation Trust, Bethlem Royal Hospital, Monks Orchard Road, Beckenham, Kent BR3 3BX UK; 4 Maudsley Learning, South London and Maudsley NHS Foundation Trust, Bethlem Royal Hospital, Monks Orchard Road, Beckenham, Kent BR3 3BX UK; 5 Buckinghamshire New University, Queen Alexandra Rd, High Wycombe, HP11 2JZ UK; 6 ESRC Centre for Society and Mental Health, King’s College London, London, WC2B 6NR UK

**Keywords:** VR; Simulation; Psychiatry; Psychology; Nursing; Experiential learning; Teaching; Restrictive practices; Stigma [3-10]

## Abstract

**Background:**

Mental health service users report that staff empathy is key to developing positive therapeutic relationships but promoting empathy in staff training is challenging. Staff may struggle to maintain their compassion, particularly in challenging settings, and have limited clinical confidence when treating conditions of which they lack subjective understanding. Novel interventions are required to address these needs.

**Main body of the text:**

Virtual reality-based simulation training has been shown to be an effective training modality for healthcare professionals; it has the potential to deliver crucial empathy-building learning for frontline mental health staff due to its capacity to increase staff understanding of service users’ experiences. Virtual reality and simulation technology take interactivity and experiential learning to a level beyond which we have seen in teaching and training before. Subjective understanding is elicited because this is a technology for enhanced experiential learning, which in turn fosters greater empathy and compassion. Increased empathy in the workforce is likely to yield significant benefits for service users. Greater empathy in nursing is linked with reduced restrictive practices and reduced conflict between staff and service users. Restrictive practices, including restraint and seclusion, are widely used in mental health settings within the UK, and are an aspect of mental health nursing that is at odds with the therapeutic role of nursing. Despite these innovative developments, there are challenges ahead. Many nurses feel that complete eradication of restrictive practices is impossible and that barriers include a limitation of resources, communication, management, and lack of education. There is a need to make simulation training economically viable so that it can be upscaled and widely available. Therefore, greater investment and resources are needed to bring this innovative training to the wider workforce to support staff and to realise the benefits for service users.

**Short conclusion:**

Virtual reality-based training has great potential for mental health staff, which could have important consequences in terms of improved staff empathy and reductions in harmful restrictive practices. Further research and funding for such training is necessary so that it can be more widely available.

## Background

Mental health service users report that staff empathy is key to developing positive therapeutic relationships [1, 2]. Empathy has been defined as understanding another person’s experience from their frame of reference [3]. While empathy is a crucial attribute for clinicians [4], promoting empathy in staff training programmes is challenging [5]. Psychiatric staff can experience role tensions when negotiating the aims of providing person-centred trauma-informed care, ensuring safety, and managing risk [6]. Staff can struggle to maintain their compassion, particularly when working in difficult settings with service users who present with complex and challenging behaviours, which can lead to burnout and reduced empathy [6]. Furthermore, psychiatric staff have limited clinical confidence when treating conditions of which they lack subjective understanding, such as hearing voices [7]. Novel interventions are required to address these needs. The Topol Review argued that virtual reality (VR) is an innovative technology that will enable diverse ways of working and staff training in health and social care [8]. The aim of this article is to highlight the merits of VR-based simulation training for mental health staff as a way of building empathy.

### Virtual reality training in mental health settings

In VR-based simulation training, trainees wear a head-mounted display or headset to experience virtual environments and these devices and environments are an integral part of the learning experience [9]. Virtual environments offer a way for trainees to observe or practice certain techniques in three-dimensional simulations that feel realistic and immersive. Until recently, head mounted displays were connected to a computer, but many contemporary models are wireless and have environments programmed into the device. This allows devices to be portable and training to be delivered more flexibly. There are clear benefits of VR-based simulation training. Evidence indicates it is convenient, engaging, and interactive [10]. VR has been shown to be effective across a range of healthcare settings and has the potential to deliver crucial empathy-building learning for frontline mental health staff due to its immersiveness, which provides direct and realistic, first-hand experiences [11, 12].

In mental health settings, simulation training has been shown to be an effective training modality for healthcare professionals because it enables staff to understand service user experience [13–19]. Benefits have been reported from involving service users and their experience in such training [20]. UK organisations such as Maudsley Simulation [21] have led the way in developing and delivering immersive educational experiences to promote recovery and empathy, while integrating de-escalation skills in VR scenarios [22]. Therefore, there is a need and an opportunity to harness VR technology that can build empathy, confidence, and compassion in the workforce, leading to these benefits for service users.

### Reduced restrictive practices

Greater empathy in nursing is linked with reduced restrictive practices in inpatient services and reduced conflict between staff and service users [23, 24]. Restrictive practices, including restraint and seclusion, are an aspect of mental health nursing that is at odds with the therapeutic role of nursing and core nursing values of compassion, respect, and kindness [25]. Restrictive practices are widely used in mental health settings within the UK. In 2015–2016, there was a 17% increase in the use of physical restraint [26]. The use of restrictive practices frequently results in serious injuries for service users and staff [27–32]. However, the new Mental Health Act White Paper highlights the need for the least restriction and greater choice and autonomy for service users [33].

Increasing staff empathy will be key to operationalise these changes. In 2015, the mental health charity Mind called for training by service users which promotes empathy and compassion, to enable staff to imagine how it feels to be restrained [31]. VR-based training could take this a step further by enabling staff to experience restrictive practices from a service user perspective, whist reinforcing existing skills which have been shown to reduce the incidences of restraint [34]. This indicates that if psychiatric staff can be empowered by VR-based training to develop greater confidence and make person-centred decisions that reduce restrictive practices, then we are likely to see significant benefits to the provision of care for service users.

### Learning theory, empathy, and stigma reduction

VR technology is uniquely placed to achieve greater empathy due to its capacity to increase staff understanding of service users’ experiences. The immersiveness of VR transports the user into the position and perspective of another individual; and evidence from Learning Theory explains why this will increase the power of VR to develop learning. Both Social Cognitive Theory and Situated Learning have been cited as key factors in support of VR-based learning [35]. The immersive, first-person perspective of VR optimises opportunities for learning, according to Social Cognitive Theory, because this learning benefits from observation and emulation in a social environment, while the realistic and authentic VR environments invoke situated learning, where the learning benefits from taking place in a particular situation. It is well-established that staff training and teaching are most effective when they incorporate experiential learning techniques [36] because this facilitates service user-centred thinking far more than didactic teaching and knowledge transfer. Typically, experiential learning has involved interactive teaching and opportunities for students to try out techniques in activities, such as roleplays. The 360-degree perspective and immersiveness of VR takes interactivity and experiential learning further by enabling learners to virtually step into service users’ shoes. Subjective understanding is elicited precisely because this is technology for enhanced experiential learning; and this in turn leads to greater empathy and compassion [37].

VR and simulation technology can contribute to stigma reduction in mental health settings. Technology that simulates psychotic experiences may reduce the so-called “them-and-us” distinction between staff and service users [38]. Simulations of hallucinations, such as service users’ experiences of hearing voices, have been shown to increase subjective understanding and empathy across a range of settings [39]; they can improve compassion in the public [40, 41] and can increase confidence in clinicians [2].

### Addressing the challenges

Despite these innovative developments, it is vital that we consider potential challenges and risks. Nurses and other healthcare professionals face obstacles to reduce restrictive practices. Many nurses feel that complete eradication of restrictive practices is impossible [6] and barriers include limited resources, communication, management, and lack of education [42–44]. Equally, while VR-based simulation has been shown to be effective in enhancing empathy, it can be resource-intensive and not easily scalable [45]. More VR-specific barriers include the initial costs, which although much reduced nowadays (e.g., £300–£700 for many VR headsets), will still be inaccessible for many healthcare services, despite evidence of longer-term cost-saving [46]; and the mild ‘cybersickness’ that can be experienced by some people who experience VR, although this is improving with contemporary lighter-weight head-mounted displays [47]. Positive user experience is a vital component of VR-based training, so it is important that all sessions involve clear face-to-face briefing and debriefing by training facilitators that explain the technology, are specific to the virtual experience, and ensure psychological and physical safety.

As the NHS is already resource-constrained, there is a need to make VR-based simulation training economically viable so that it can be upscaled and widely available. Therefore, greater investment and resources are needed to bring this innovative training to the wider workforce to support staff and benefit service users.

## Conclusions

VR-based training has great potential for mental health staff because of the way that it increases subjective understanding of service user experience. This could have important consequences in terms of improving staff empathy towards service users and the potential to reduce harmful restrictive practices, which should be a key outcome for future VR training in mental health to evaluate. Further research and funding for such training is therefore necessary so that VR-based simulation training can be more widely available.

## Data Availability

Not applicable.
